# Experimental quantum secure direct communication with single photons

**DOI:** 10.1038/lsa.2016.144

**Published:** 2016-09-09

**Authors:** Jian-Yong Hu, Bo Yu, Ming-Yong Jing, Lian-Tuan Xiao, Suo-Tang Jia, Guo-Qing Qin, Gui-Lu Long

**Affiliations:** 1 State Key Laboratory of Quantum Optics and Quantum Optics Devices, Institute of Laser Spectroscopy, Shanxi University, Taiyuan, Shanxi 030006, China; 2 Collaborative Innovation Center of Extreme Optics, Shanxi University, Taiyuan 030006, China; 3 State Key Laboratory of Low-Dimensional Quantum Physics and Department of Physics, Tsinghua University, Beijing 100084, China; 4 Collaborative Innovation Center of Quantum Matter, Beijing 100084, China; 5 Tsinghua National Laboratory for Information Science and Technology, Tsinghua University, Beijing 100084, China

**Keywords:** block transmission, channel loss and noise, DL04 protocol, quantum secure direct communication, single-photon frequency coding

## Abstract

Quantum secure direct communication is an important mode of quantum communication in which secret messages are securely communicated directly over a quantum channel. Quantum secure direct communication is also a basic cryptographic primitive for constructing other quantum communication tasks, such as quantum authentication and quantum dialog. Here, we report the first experimental demonstration of quantum secure direct communication based on the DL04 protocol and equipped with single-photon frequency coding that explicitly demonstrated block transmission. In our experiment, we provided 16 different frequency channels, equivalent to a nibble of four-bit binary numbers for direct information transmission. The experiment firmly demonstrated the feasibility of quantum secure direct communication in the presence of noise and loss.

## Introduction

Secure communication is not only vital in military use and national security, but also important in modern everyday life. Quantum communication provides a novel way of communication with unconditional security. The fundamental difference between quantum communication and classical communication is on the capability to detect eavesdropping on-site. There are different modes of quantum communication: quantum key distribution (QKD)^1^, quantum secret sharing^2^, quantum secure direct communication (QSDC)^3^, quantum teleportation^4^ and quantum dense coding^5^.

Since the earliest BB84 protocol was proposed^1^, QKD has been researched extensively, and the application over a distance of a few hundreds of kilometers has been achieved^6^. QKD can be completed non-deterministically, for instance, in the BB84 and BBM92 protocols^1, 7^, where the key is distributed indeterminately. Alternatively, deterministic QKD communication^8, 9, 10, 11, 12, 13^ is essentially a deterministic QKD process plus a classical communication. Alice first chooses a random key and uses it to encrypt the secret message into ciphertext, and then transmits the ciphertext to Bob through a quantum channel. If both of them are certain that no eavesdroppers exist, Alice sends the key to Bob through a classical channel.

In contrast to QKD communication, QSDC sends secret information securely through a quantum channel directly without setting up a prior key^3, 14, 15^. Since the first QSDC protocol was proposed^3^, it has become one of the hot research topics in quantum communication over the past decade. The secure direct nature of QSDC also makes it an important cryptographic primitive. Protocols of quantum signature^16^, quantum dialog^17, 18^ and quantum direct secret sharing^19, 20^ were all constructed on the basis of QSDC. The security of QSDC relies on quantum principles, such as the no-cloning theorem, the uncertainty principle, correlation of entangled particles and nonlocality. In addition, QSDC has been enhanced by a block transmission technique that was proposed in the first QSDC protocol by Long and Liu^3^. For entanglement carriers, in 2003, Deng *et al.*^21^ proposed a two-step QSDC protocol where the criteria for QSDC were explicitly stated. QSDC protocols based on high-dimensional entanglement^22, 23, 24^, multipartite entanglement^25, 26, 27^ and hyperentanglement^28^ were developed one by one. For single photons carriers, the first QSDC protocol was proposed in Ref. 29, the so-called DL04 protocol, wherein, the information was directly encoded in the single photons. Here, 0 is encoded with *I*=|0〉〈0|+|1〉〈1| and 1 with *U*=*iσ*_*y*_=|0〉〈1|−|1〉〈0|. High-capacity QSDC protocols were proposed with single photons carriers^30^, which can carry 2 bits of information with a single photon, as the sender encodes the message in both the polarization state and the spatial-mode state, independently.

However, the channel loss of the photons would lead to the loss of the secret information when it is encoded in the individual photons. When there is noise in the quantum channel, an adversary Eve can gain a certain amount of information by hiding her presence in the channel noise. In this case, the information leakage may be eliminated by using either quantum error correction^31^ or quantum privacy amplification^32^. Unfortunately, quantum privacy amplification ruins the direct communication picture because it involves merger and order reshuffling of single photons. An efficient way to implement QSDC in a noisy channel is to use quantum error correction^31, 33^. Post-processing can be performed using quantum error correction without using privacy amplification and reconciliation^34^. In this work, instead of using the complicated quantum error correction, we present a new QSDC protocol on the basis of a single-photon frequency coding scheme, called the FRECO-DL04 protocol. The information is encoded in the frequency spectrum of a block of single photons rather than on the individual photons. It is experimentally shown that FRECO-DL04 can work efficiently in the presence of channel loss and noise.

## Materials and methods

### FRECO-DL04 protocol

Suppose that Bob wants to send secret information to Alice. The protocol contains the following four steps:

1. Alice prepares a block of *N*_2_ single photons. Each photon in the block is randomly in one of four states: |0〉, |1〉, |+〉 and |−〉, where |0〉 and |1〉 are the eigenstates of the Pauli *Z* operator, and |±〉=(|0〉±|1〉)/√2 are the eigenstates of the Pauli *X* operator. Then, Alice sends the single-photon block to Bob, and Bob acknowledges this fact.
2. Because of channel noise and loss, Bob receives only *N*_1_ single photons (*N*_1_<*N*_2_). He selects *CN*_1_ number (*C* is a positive number less than or equal to 1/2) of photons randomly from the *N*_1_ received photons for eavesdropping check by measuring them randomly in the *X*-basis or the *Z*-basis (control mode^29^). Then, Bob tells Alice the positions, the measuring-basis and the measuring results of these measured photons. Alice compares her results with those of Bob and obtains an error rate. If the error rate is higher than the threshold, they abort the communication. If the error rate is less than the threshold, the Alice-to-Bob communication is considered safe and continues to step 3.
3. The remaining (1−*C*)*N*_1_ received photons are used for encoding the secret information (Encode mode). Bob also selects *C*(1−*C*)*N*_1_ single photons from the remaining photons randomly as check bits for the Bob-to-Alice transmission and randomly applies one of the two operations, *U*=*iσ*_*y*_=|0〉〈1|−|1〉〈0| and *I*=|0〉〈0|+|1〉〈1|, which flips or does not flip the state of the photon. The rest of the single photons are processed by the single-photon frequency coding scheme, which are described below.
4. Bob sends the encoded photon block back to Alice who can deterministically decode Bob’s operations by measuring the photons in the same basis as she prepared them. Alice obtains the operation of each single photon in the block and their arrival time. Because of channel loss, Bob receives only *N* (here *N*≤(1−*C*)^2^*N*_1_) photons in each block after subtracting the check photons. Alice and Bob also publicly compare the results of the checking bits to check for eavesdropping in the Bob-to-Alice transmission. Next, Alice analyzes the frequency spectrum and determines Bob’s encoded bits and retrieves the secret information.

### Single-photon frequency coding

In the DL04 protocol, the information is directly encoded in the individual photons, where 0 is encoded with operation *I* and 1 with *U*. The operation *U* flips the state without changing the measurement basis, namely


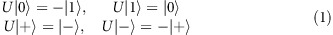


Instead of using an individual operation to encode a bit value, single-photon frequency coding applies a series of operations periodically on a single-photon block to encode information. Bob applies the operations *U* and *I* on single photons in the block according to a periodic function with period *T*=1/*f*, where *f* is the modulation frequency that encodes the information. Typically, different modulation frequencies correspond to the different binary bit sequences. Once Alice obtains the modulation frequencies spectrum after she measures a block of single photons, she gets Bob's information fully. The encoding operation Bob applies to the single-photon block, after excluding the checking bits, is


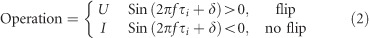


where *δ* is the initial phase of each modulation signal, which could be an arbitrary value between 0 and 2*π*, and *f* is the modulation frequency. An example is given in Table 1, where the initial states, the final states, the measured operations *x*_(*i*)_ and arrival times *τ*_*i*_ are shown. The measured values *x*_(*i*)_ that Alice obtained denote Bob’s flip *U* (denoted as 1) or no flip *I* (denoted as 0) operations. Alice records the arrival time *τ*_*i*_, for *i*=1, 2, 3,…, *N*, where *N* is the number of single photons that she has measured in each block after subtracting the check photons.

Not all the photons can arrive at Alice’s side because of the loss of optical fiber and imperfect detection efficiency of the single-photon detector. However, this single-photon frequency coding scheme is robust against loss and error. The information is encoded in the frequency spectrum of the single-photon block, instead of individual photons, where the loss and error of some photons would change only the signal-to-noise ratio (SNR) of the frequency spectrum. The modulation frequency can be accurately determined from the block (*x*_(*i*)_, *τ*_*i*_) using the discrete time Fourier transform,


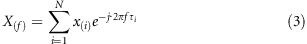


From the frequency spectrum line at the modulation frequency, Alice can determine the encoded frequency and reads out the secret information.

For a given quantum communication system, there exists a finite maximum number *N*_c_ of frequency channels,


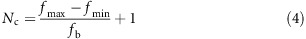


where *f*_max_ and *f*_min_ are the maximum and minimum modulation frequencies, respectively, and *f*_b_ is the channel spacing. The information transmission capacity relies on the number of frequency components. Assuming Bob loads *r* frequency components on one single-photon block, the effective degrees of freedom are the total number of different combinations of *r* frequencies over the *N*_c_ frequency channels,


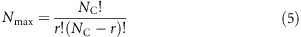


which means one single-photon block can carry *b*=log_2_*N*_max_ bits of information. The transmission rate can be expressed as


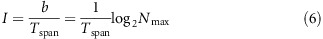


where *T*_span_ is the time span, that is, the time length of a single-photon block. The principle of the coding scheme is similar to the ultra-wide-band communication in the field of wireless communication^35^.

### Experimental setup

**Figure 1:**
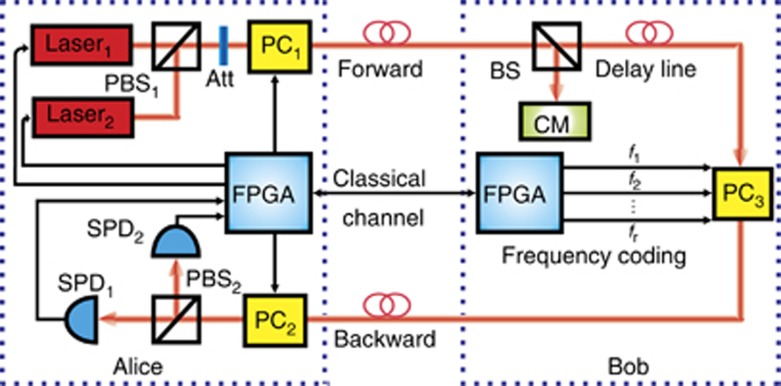
Schematic diagram of the experimental setup of the FRECO-DL04 protocol. PBS, Polarization beam splitter; Att, Variable attenuator; PC, Polarization controller; BS, Beam splitter; CM, Control mode; FPGA, Field programmable gate array; SPD, Single-photon detector. The distance between Alice and Bob is *L*_1_, and the delay line length is *L*_2_.

The experimental setup is shown in Figure 1. A strong attenuated laser (1550 nm, NP Photonics RELS) was used as a single-photon source with systematic pulse repetition frequency of 10 MHz. Alice sends the single-photon block to Bob. The QSDC operation system is controlled by a field programmable gate array (FPGA) device. The control mode, as shown in Figure 1, is used to check for eavesdropping. Bob randomly selects a subset of the received photons after the beam splitter. For those photons that Bob measured, he records the photons’ arrival times. Therefore, both Alice and Bob knew the arrival time of the pulses. They compare the measured results and calculate the error rate to check with the threshold. The encoding operation, that is, the polarization flip operation of the four states of single photons is realized using the two serially aligned electro-optical modulators (EO-AM-NR-C3)^36^. The optical axis of the two modulators is adjusted to a 45° angle. The single photons are detected using a single-photon detector (QCD300). During the eavesdropping detection procedure of the block, an optical fiber (with length *L*_2_) is used as a delay line to synchronize the encoded photons.

In our experiment, the highest modulation frequency *f* is limited by the time jitter of the single-photon detector, the computing rate of the microprocessor and the frequency response of the modulator. The channel spacing is determined by the full width at half maximum of the characteristic spectrum, which depends on the length of the photon block and the mean photon count per pulse. Here, the channel spacing is 25 kHz, which is determined by the 80 end-detected photon counts and 1 ms block time, and could be smaller under the increscent photon counts and block time.

## Results and discussion

**Figure 2:**
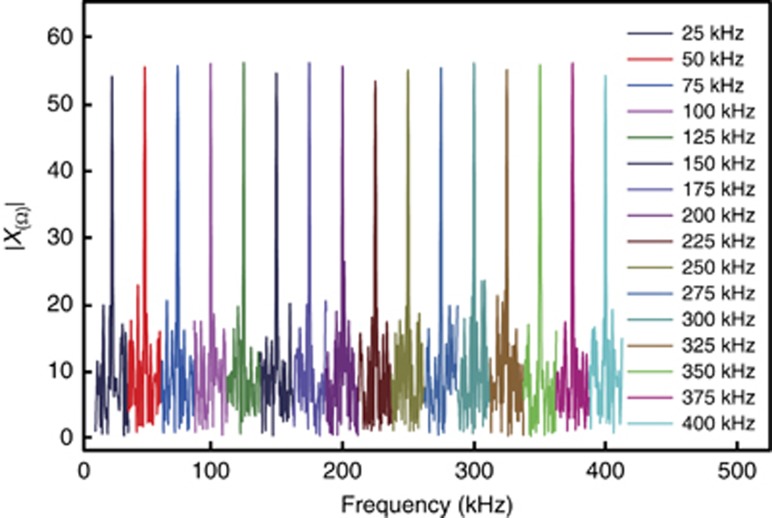
The experimental results of the modulation frequency spectrum. The *y*-axis is the Fourier-transformed amplitude in Equation (3). The different color lines represent different modulation frequencies. These 16 modulation frequency spectrum lines correspond to binary numbers from 0000 to 1111. The systematic pulse repetition frequency is 10 MHz.

**Figure 3:**
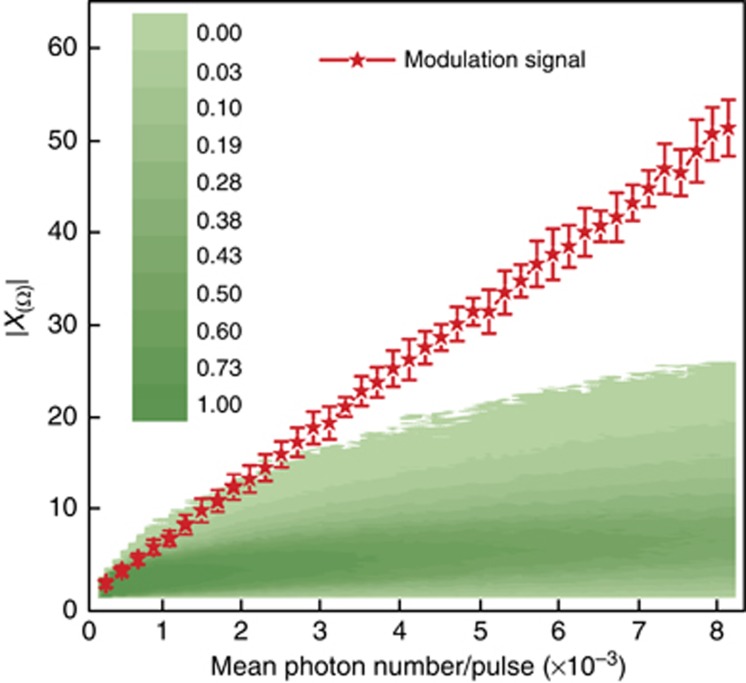
The characteristic spectrum and background noise distribution of the modulation frequency spectrum. The *x*-axis is the mean photon count per pulse that Alice detects. The green colored areas are the background white noise in the experiment, where the color depth represents the relative probability distribution of the noise. The red line represents the amplitude of the characteristic spectrum. The modulation frequency is 200 kHz.

The results of the spectral analysis of the block (*x*_(*i*),_ *τ*_*i*_) using Equation (3) are shown in Figure 2. There is a white noise background in the frequency spectrum because the photon number of coherent light pulses obeys a Poisson distribution. There is a characteristic spectrum at the modulation frequency above the white noise background, which enables Alice to retrieve the information encoded by Bob. The noise and loss of the quantum channel decrease the SNR of the characteristic spectrum. Figure 3 shows the relationship between the signal and background noise with different mean photon numbers. With a relative larger photon number per pulse, the amplitude of the characteristic spectrum is higher than the background noise. Furthermore, our previous work^37^ showed that the SNR does not change with the modulation frequency. In our frequency-coding experiment, we take *N*_c_=16 frequency channels from 25  to 400 kHz with channel spacing 25 kHz. Using a onefold frequency component *r*=1, which means only one of the 16 frequency channels will be used for information transmission in one time span, Alice can get log_2_16=4 bits of information by processing one block of data in one time span. When the length of the block is 1 ms, the transmission rate reaches 4 kbps. A detailed example of the nibble of four-bit binary numbers is given in the Supplementary Information.

### The security analysis of the FRECO-DL04 protocol

Here, we consider two common eavesdropping strategies: intercept-resend attack and photon-number-splitting attack. When the single-photon source is an attenuated laser with mean photon number *μ* per pulse, the probability *p*_n_ to have *n* photons in a single pulse follows a Poisson distribution. The probability that an optical pulse could be detected at the receiving end is^38^


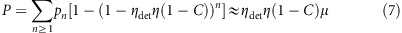


where $\eta =10^{-\alpha (2L_{1}+L_{2})/10}$ is the optical attenuation due to the loss of the fiber (the total fiber length is 2*L*_1_+*L*_2_); *L*_1_ and *L*_2_ are the communication distance and the fiber length of the optical delay line at Bob’s side, respectively. *α* is the optical fiber loss coefficient (typical value is 0.2 dB km^−1^), and *η*_det_ is the quantum efficiency of the single-photon detector^39, 40^. Equation (7) is valid if *η*_det_*ηp*_n_*n*«1 for all *n*.

With multi-photon pulses, Eve performs a photon-number-splitting attack. First, she performs a quantum non-demolition measurement on the pulses as soon as they exit Alice's station. When *n*=2, Eve stores one photon *P*_1_ and sends the other one *P*_2_ to Bob using a lossless channel. After Bob's encoding operation, Eve captures the photon again. To gain Bob’s secret information, Eve must judge whether the polarizations of the two photons is parallel or antiparallel^41^. However, there is no measurement strategy for Eve to determine whether the photon *P*_2_ is flipped by Bob. Therefore, no information can be obtained by Eve from the two-photon pulses. When *n*=3, there is a measurement *M* that provides a conclusive result about whether the polarization is flipped with a probability 1/2 (Ref. 42). When *n*>3, we assume that Bob can always judge whether the polarization is flipped conclusively. For pulses with three or more photons, she executes *M*; if the outcome is not conclusive, she blocks these pulses, but if the outcome is conclusive, she prepares a new photon in the same state and forwards it to Bob. After Bob's encoding operation, Eve measures the photon again on the backward trip to see whether the polarization state has been flipped. From these operations, the mean amount of effective qubits per pulse that Eve can get is


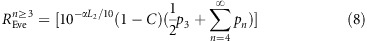


Both eavesdropping strategies do not cause any bit error, which means that Eve cannot be detected during such an eavesdropping process.

In a noisy channel, when *n*=1, Eve performs the intercept-resend attack. She may gain a certain amount of data without being detected by hiding her presence in the noise if she replaces the noisy channel by an ideal one and sends another photon prepared by herself to Alice. She could acquire a fraction 4*e* of the qubits on the forward Alice–Bob channel, where *e* is the bit error rate caused by channel noise. Factor 4 arises because there is a 50% chance for Eve to pick the correct basis, but when she picks the wrong basis, there is a 50% chance of not causing a bit error. The mean effective qubits per pulse that Eve can get is


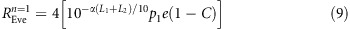


Considering all the strategies, the mean amount of effective qubits per pulse that Eve eventually gets is


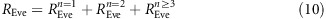


**Figure 4:**
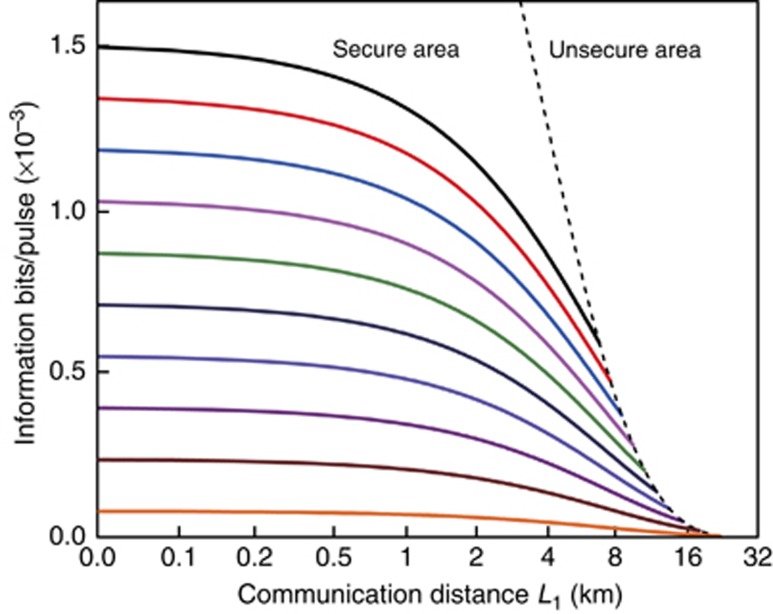
The calculated transmitted information bit per pulse versus the communication distance. The dotted line is the cut-off line of the secure communication area. The solid lines with different colors represent different mean photon numbers per pulse (*μ*=0.19, 0.17, 0.15, 0.13, 0.11, 0.09, 0.07, 0.05, 0.03, 0.01, from top to bottom). Here, *η*_det_=0.32, *e*=5‰, *α*=0.2 dB km^−1^, *L*_2_=*L*_1_, and *C*=1/2.

The number of qubits that Alice gets and the transmission rate of Alice, respectively, can be derived from Equation (7)


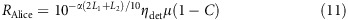



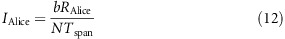


where *b* is the bit-string length of the secret information encoded in a single-photon block, and *N* is the number of single photons that Alice detects within the time span *T*_span_. The SNR of the characteristic spectrum is determined by the number of correct detections of the encoded single photons. Although Eve may acquire some photons of the encoded single-photon block conclusively, it is impossible for her to get all secret information bits when the SNR is <1. The secure information bits per pulse and secure communication distance are shown in Figure 4. Here, we set *η*_det_=0.32, *e*=5‰, *α*=0.2 dB km^−1^, *L*_2_=*L*_1_ and *C*=1/2. The secure communication distance relies on the mean photon number per pulses. Typically, for weak laser pulses with mean photon number 0.1 per pulse, the secure distance is ~10 km.

## Conclusions

In summary, we presented a new practical QSDC protocol, the FRECO-DL04 protocol, on the basis of the DL04 protocol equipped with single-photon frequency coding. Instead of quantum error correction procedure, in our protocol, the information is encoded in the modulation frequency spectrum of the single-photon block. We demonstrated the FRECO-DL04 protocol experimentally, which is the first time the block transmission has been demonstrated. With a onefold frequency component, we provided a nibble of four-bit binary numbers for direct information transmission using 16 different frequency channels. A transmission rate of 4 kbps has been achieved. The experiment firmly demonstrated the principle of QSDC with the presence of practical channel noise and loss. The FRECO-DL04 protocol could adopt multifold frequency components simultaneously, considerably increasing the amount of information that a block of single photons can carry.

## Figures and Tables

**Table 1:** Operations of single photons for block transmission

Initial state	↕	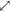	↕	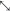	↔	…	↔
Final state	↕	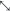	↔	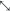	↕	…	↔
*x*_(*i*)_	0	1	1	0	1	…	0
Time *τ*_i_	*τ*_1_	*τ*_2_	*τ*_3_	*τ*_4_	*τ*_5_	…	*τ*_*N*_
