# Supplementary material for: Experimental quantum secure direct communication with single photons
Source: Light Sci Appl. 2016 Sep 9;5(9):e16144–. doi: 10.1038/lsa.2016.144 (PMC6059926; doi:10.1038/lsa.2016.144)
Supplement: Supplementary Information [file lsa2016144x1.pdf]

Supplementary Information to
Experimental quantum secure direct communication with single photons

1. An example of single-photon frequency coding

In this section, a detailed example of single-photon frequency coding used for transmitting the word “Light” is given. We use 16 frequency channels for information transmission: 25kHz, 50kHz, 75kHz, 100kHz, 125kHz, 150kHz, 175kHz, 200kHz, 225kHz, 250kHz, 275kHz, 300kHz, 325kHz, 350kHz, 375kHz, and 400kHz. These 16 frequency channels are corresponding to the binary number from 0000 to 1111, respectively. To the letter “L”, the corresponding binary sequence is [01001100] according to ASCII code. Here the nibbles of four-bit binary numbers [0100] and [1100] are corresponding to the modulation frequencies 100 kHz and 300 kHz, respectively.

The corresponding binary sequence of the word “Light” is [01001100 01101001 01100111 01101000 01110100]. The relationship of word “Light” and the modulation frequencies are listed in the following table.

Table SI1 Corresponding relationship of word “Light” and the modulation frequencies

	L		i		g		h		t	
Binary	0100	1100	0110	1001	0110	0111	0110	1000	0111	0100
Modulation frequency (kHz)	100	300	150	225	150	175	150	200	175	100

In the process of information transmission, Bob first sets the modulation frequency at 100 kHz for the first block of photons, then 300 kHz on the second block of photons for the character "L". Alice measures the frequency spectra of the two modulated single-photon blocks and determines the modulation frequencies Bob used. The information of letter “L” can then be extracted from frequency spectra, as shown in the Fig. SI1.

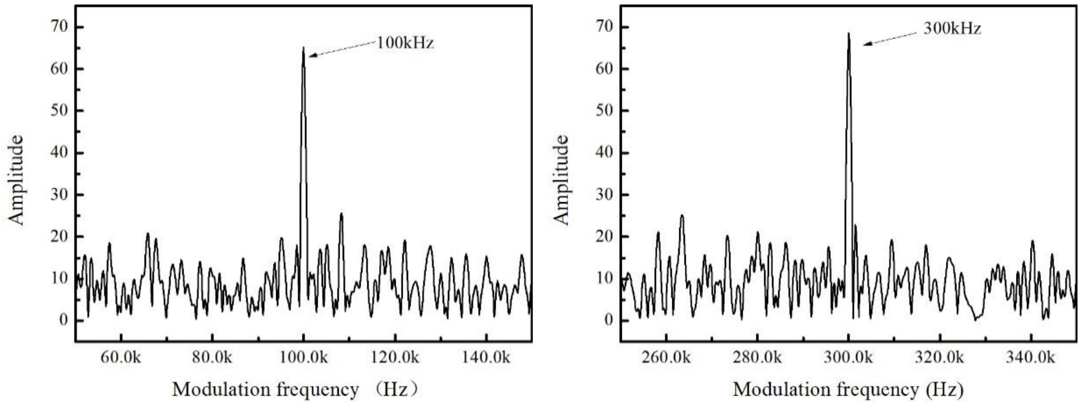

Fig. SI1. The frequency spectra of the modulated single-photon blocks. The two figures corresponding to modulation frequencies 100 kHz and 300 kHz, respectively.

Carrying on the above process with different modulation frequencies on the each of the following blocks of single photons according to Table SI1, Alice can retrieve the whole word “Light”.

In the above binary coding process, there needs only one frequency component at once. The coding

scheme can be easily extended to the multifold frequency components ($r \geq 2$) form so as to increase the capacity, just like color image can be transmitted based on three frequency components in parallel, encoding the gray levels of three primary colors.

2. Decoding process of single-photon frequency coding scheme.

2.1 The frequency spectrum of single-photon block without modulation.

For a single-photon block originating from an attenuated laser which obeys Poisson distribution in time domain, the frequency spectrum shows as white noise distribution, which can be extracted out through the discrete time Fourier transform, as show in Fig. SI2.

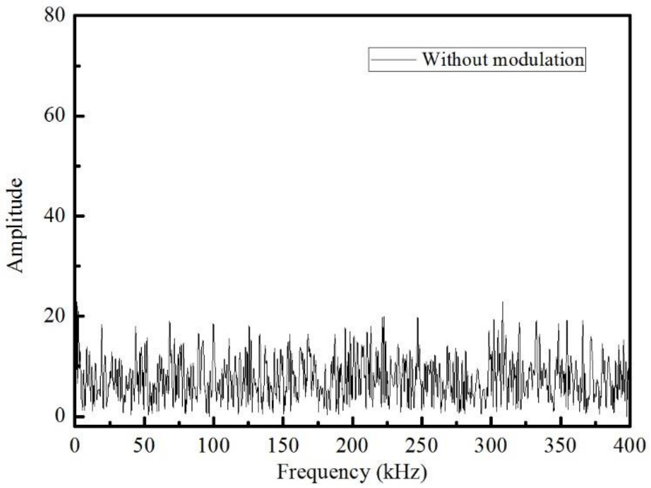

Fig. SI2. The white noise spectrum of the single-photon block without modulation. The mean photon counts is 80kcps; the time span is 1 ms.

2.2 The frequency spectrum of single-photon block with modulation.

For the modulated single-photon block, the frequency spectrum is no longer the barely white noise distribution.

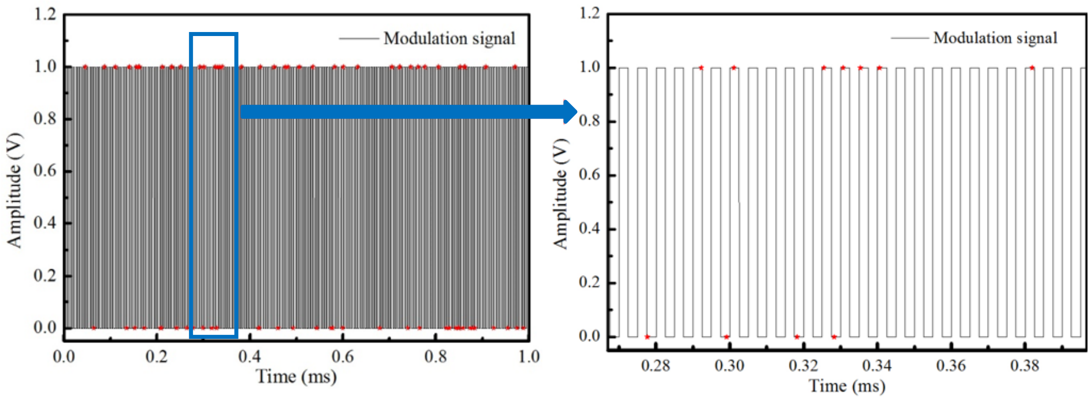

Fig. SI3. Diagram of Bob's encoding operation on the single-photon block by using the frequency modulation. Here the high (or low) level of the black line corresponding to the operation *U* (or *I*). Operation *U*, *I* correspond to flip and no flip operations on the states of the photon. The red dots are detected single-photons.

As show in the Fig. SI3, when the modulation signal is positive, Bob operates the operation $U = i\sigma_y = |0\rangle\langle 1| - |1\rangle\langle 0|$. When the modulation signal is negative, Bob operates the operation $I = |0\rangle\langle 0| + |1\rangle\langle 1|$. 80 photons randomly distribute within 1 ms, the modulation frequency is 200 kHz.

The discrete time Fourier transform is

$$X_{(f)} = \sum_{i=1}^{N} x_{(i)} e^{-j \cdot 2\pi f \tau_i}$$

Here, $x_{(i)}$ depends on the operation I or U that Bob applied. τ_i is the arrival time of the ith detected photon. Figure SI4 shows the frequency spectrum of the modulated single-photon block. Note that there is a modulation signal at the modulation frequency 200 kHz. However, to the eavesdropper, she does not know modulation operations. Therefore to her, the spectrum is still a white noise.

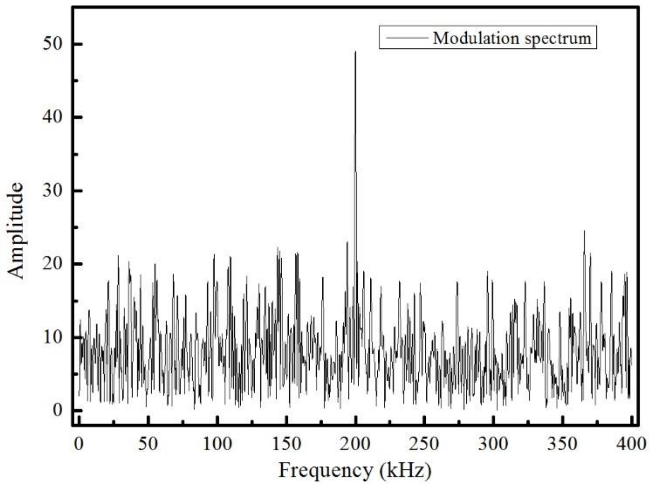

Fig. SI4. The frequency spectrum of the modulated single-photon block. The mean photon counts is 80kcps; the time span is 1 ms.
